# A Fuzzy Rule-Based System for Classification of Diabetes

**DOI:** 10.3390/s21238095

**Published:** 2021-12-03

**Authors:** Khalid Mahmood Aamir, Laiba Sarfraz, Muhammad Ramzan, Muhammad Bilal, Jana Shafi, Muhammad Attique

**Affiliations:** 1 Department of Computer Science and Information Technology, University of Sargodha, Sargodha 40100, Pakistan; khalid.aamir@uos.edu.pk (K.M.A.); laibasarfaraz1@gmail.com (L.S.); muhammad.ramzan@uos.edu.pk (M.R.); 2 School of Systems and Technology, University of Management and Technology, Lahore 54782, Pakistan; 3 Department of Computer Science, National University of Computer and Emerging Sciences, Islamabad 44000, Pakistan; 4 Department of Computer Science, College of Arts and Science, Prince Sattam bin Abdul University, Wadi Ad-Dwasir 11991, Saudi Arabia; j.jana@psau.edu.sa; 5 Department of Software, Sejong University, Seoul 05006, Korea

**Keywords:** diabetes, fuzzy logic, fuzzy rule-based system, diabetes prediction, classification

## Abstract

Diabetes is a fatal disease that currently has no treatment. However, early diagnosis of diabetes aids patients to start timely treatment and thus reduces or eliminates the risk of severe complications. The prevalence of diabetes has been rising rapidly worldwide. Several methods have been introduced to diagnose diabetes at an early stage, however, most of these methods lack interpretability, due to which the diagnostic process cannot be explained. In this paper, fuzzy logic has been employed to develop an interpretable model and to perform an early diagnosis of diabetes. Fuzzy logic has been combined with the cosine amplitude method, and two fuzzy classifiers have been constructed. Afterward, fuzzy rules have been designed based on these classifiers. Lastly, a publicly available diabetes dataset has been used to evaluate the performance of the proposed fuzzy rule-based model. The results show that the proposed model outperforms existing techniques by achieving an accuracy of 96.47%. The proposed model has demonstrated great prediction accuracy, suggesting that it can be utilized in the healthcare sector for the accurate diagnose of diabetes.

## 1. Introduction

Diabetes mellitus (DM) is considered chronic disease in which the required amount of insulin is not produced by the body or insulin is not properly used by the body, resulting in excessively high blood sugar (glucose) levels [1]. The number of people affected by diabetes in 2015 was 415 million. This number is predicted to surpass 642 million by 2040. Moreover, the prevalence of undiagnosed diabetic patients is up to 179 million [2]. Furthermore, according to the World Health Organization (WHO), diabetes caused 4.6 million deaths in 2011, and it will be the seventh major cause of mortality by 2030. The number of diabetic patients has been increased with every passing year and becoming a challenge for the healthcare sector. Early diagnosis of diabetes has been improved by recent advances in the healthcare sector, but approximately half of the patients are not aware of their ailment. It can take more than 10 years to diagnose them. Serious health complications such as kidney failure, risk of blindness, blood pressure, nerve damage, and stroke can develop with treatment delay. Diabetes is currently an incurable disease, and its treatment efficiency is primarily dependent on accurate diagnosis and timely treatment. If diabetes is detected in its initial phase, then the disease can be controlled. On the other hand, if diabetes is left undetected or untreated, it can cause serious harm to the body and make it difficult to treat, while early diabetes detection can lead to better treatment, resulting in lower morbidity and deaths.

In order to detect diabetes, a wide variety of technologies and algorithms have been employed by researchers during the past few years. Machine learning (ML) is one of these technologies. During this fourth industrial revolution, machine learning has been proved a valuable tool in various areas including, healthcare [3,4,5]. Artificial intelligence (AI), data mining, neural networks (NN), and many others are considered essential branches of ML that are crucial in the healthcare sector, specifically in diabetes detection [6]. However, while most of these technologies can be used to predict diseases accurately, their designs and reasoning processes are often not interpretable, making them difficult to understand and they are therefore considered as “black boxes”. The process of disease detection and data inference can’t be explained using machine learning technologies [7]. Therefore, it is crucial to employ technologies that are interpretable and understandable to humans. Moreover, another drawback of these technologies is that they cannot deal with the vagueness of data.

Fuzzy logic was developed to address these issues. It was first introduced by Zadeh [8]. It is considered as the extension of Boolean logic in which values lie between 0 and 1, which is called the degree of membership (belongingness). Fuzzy logic is analogous to human thinking systems. Therefore, it can be used to handle the vagueness present in data. By permitting overlapping class definitions and having powerful capabilities to manage ambiguity and vagueness, fuzzy logic has proven a valuable tool for classification problems. Moreover, the use of fuzzy rule-based systems (FRBS), which employ if-then rules, improves interpretability and gives more insight into the classifier structure [9]. Furthermore, an object can be assigned to several classes with different degrees of membership. FRBS is easily interpretable by humans as they are represented in linguistic forms compared to machine learning technologies [10,11]. These characteristics have made fuzzy logic a useful technique for the accurate and early prediction of diabetes. Therefore, serious complications of the disease can be avoided.

The primary objective of this research is to identify diabetes in its early stages so that patients can receive prompt treatment and prevent the severe complications linked with this deadly disease. Moreover, this research has intended to provide high classification accuracy. Furthermore, complicated data has not been required in this study to predict diabetes; instead, it employs simple features such as age, BMI, and others to predict diabetes. In this paper, FRBS has been used to early predict diabetes using features such as blood glucose level, body mass index (BMI), skin thickness, diabetes pedigree function, age, etc. The performance of the entire system has been evaluated using a diabetes dataset. The proposed FRBS has yielded good results, indicating that it can predict diabetes with greater accuracy than previous methods.

This study plays an important role in the research era regarding the early detection of diabetes. It has provided great classification accuracy in predicting diabetes. As compared to other studies that employ fuzzy logic (FL), the proposed study has achieved the highest classification accuracy.

The rest of the paper is divided as follows: A literature review about the latest advancements in the field of diabetes detection is given in Section 2. The methodology of this research is presented in Section 3. A discussion about the results obtained from the proposed methodology is included in Section 4. A comparative analysis of the obtained results is also included in Section 4. Section 5 concludes this research by highlighting the problem area and also discussing the importance of this work.

## 2. Related Work

Several researchers have been employed machine learning (ML) techniques and fuzzy logic (FL) to predict diabetes using different diabetes datasets. In this section, we have included only those studies that used datasets similar to our dataset. Our dataset has eight attributes and 768 entries. All the entries are women and at least 21 years old.

### 2.1. Machine Learning Techniques for Diabetes Detection

This section includes ML techniques for diabetes detection. Table 1 summarizes all the papers discussed in this section. Sisodia et al. [12] conducted an experiment to detect diabetes in patients with high classification accuracy. Naive Bayes (NB), decision tree (DT), and support vector machine (SVM) were the three machine learning classification techniques used by the researchers. However, feature selection methodologies were not used to extract the features; instead, the K10 protocol was used. To test these classification algorithms, the Pima Indians Diabetes (PID) dataset was used. With an accuracy rate of 76.30%, the NB classifier outperformed the SVM and the DT. In another study, Naz et al. [13] conducted research on different machine learning classification techniques with the aim of diabetes prediction. Artificial neural network (ANN), NB, DT, and deep learning (DL) were compared. To evaluate the performance of classifiers, a diabetes dataset was used. When compared to other classifiers, DL attained the highest accuracy (98.07%). The accuracy could be improved further by using omics data. Similarly, Khanam et al. [14] compared several machine learning techniques to predict diabetes in its early stages. The researchers used a diabetes dataset to evaluate the performance of several algorithms. Seven ML techniques: SVM, random forest (RF), logistic regression (LR), AdaBoost (AB), DT, k-nearest neighbours (kNN), and neural network (NN) were used. LR and SVM performed well, while neural network (NN) outperformed the other techniques and achieved an accuracy of 88.6% on the Pima Indians Diabetes (PID) dataset.

Hasan et al. [15] predicted diabetes using a weighted ensemble model based on different ML classifiers (KNN, RF, AB, DT, NB, and XGBoost) and multilayer perceptron (MLP). To calculate the weights of each ML classifier, the area under the ROC curve (AUC) of the classifier was used. A diabetes dataset was used to check the performance of the proposed model. The results demonstrated that the proposed ensemble classifier achieved 78.9% sensitivity, 93.4% specificity, and 95% AUC. Moreover, it outperformed many state-of-the-art studies by 2.0% percent in AUC. Singh et al. [16] proposed an ensemble model, called eDiaPredict, for the prediction of diabetes. They combined a variety of machine learning approaches, including DT, RF, SVM, XGBoost, and NN. Several performance matrices were employed to test the performance of the ensemble model. Lastly, a diabetes dataset was used to evaluate the performance of the model. The model demonstrated an accuracy of 95%.

Pradhan et al. [17] suggested an artificial neural network (ANN) model for detecting diabetes in patients. The Pima Indians Diabetes (PID) dataset was used to test the working of the proposed model. The data normalization was performed in the data preprocessing stage. Afterward, training data was used to train the ANN. Finally, the model performance was evaluated using testing data. With 70% training data and 30% testing data, the model achieved an accuracy of 85.09%. In another study, Kannadasan et al. [18] proposed a deep neural network (DNN) classifier to predict diabetes. A stacked autoencoders approach was used to extract the optimal features and a SoftMax layer to classify diabetes. Moreover, the neural network was fine-tuned using backpropagation. Furthermore, the PID dataset was used to train and test the performance of the classifier. The results demonstrated that the classifier achieved an accuracy of 86.26%.

Azad et al. [19] proposed a model PMSGD to classify diabetes. Synthetic minority over-sampling technique (SMOTE), genetic algorithm (GA), and DT were used in the proposed model. The proposed model was constructed using four layers. In the first layer, data preprocessing was performed. Optimal features for training were chosen in the second layer. The model was trained in the third layer. In the fourth layer, model performance was evaluated using different performance matrices. The model was tested on the Pima Indians Diabetes (PID) dataset and achieved an accuracy of 82.1256%. Kumari et al. [20] proposed a reliable diabetes classification and prediction model (DCPM). At first, the data was preprocessed. Then, using kNN, the best value for k was determined and the model was trained using the k value. Finally, the model’s performance was assessed using various performance matrices. The model achieved an accuracy of 92.28% on the PID dataset. Abokhzam [21] et al. proposed a method to predict diabetes using an ML grid-based RF classifier. The framework was divided into two phases: training and testing. The training phase included data pre-processing, optimal feature selection, and model training. The testing phase included data pre-processing, optimal feature selection, and diabetes prediction. The researchers used the diabetes dataset to evaluate the performance of the model. The model achieved an accuracy of 95.7%. Furthermore, the researchers incorporated natural language processing with the model.

**Table 1:** Summary of machine learning techniques for diabetes detection.

Sr. No.	Reference	Year	Methodology	Finding and Results
1	Sisodia et al. [12]	2018	Naive Bayes, SVM, and DT	NB classifier outperformed the other classifiers with an accuracy of 76.30%.
2	Naz et al. [13]	2020	Artificial Neural Network (ANN), Bayes, Decision Tree, and Deep Learning	Deep Learning (DL) attained the highest 98.07% accuracy
3	Khanam et al. [14]	2021	SVM, DT, k-Nearest Neighbours (kNN), Random Forest (RF), Logistic Regression (LR), AdaBoost (AB), and Neural Network (NN)	Neural Network (NN) outperformed the other techniques and reached an accuracy of 88.6% on the Pima Indians Diabetes (PID) dataset
4	Hasan et al. [15]	2020	Weighted ensemble model of kNN, DT, RF, AB, NB, and XGBoost	The results demonstrated that the proposed ensemble classifier achieved 78.9% sensitivity, 93.4% specificity, and 95% AUC
5	Singh et al. [16]	2021	Ensemble model of DT, RF, SVM, XGBoost, and NN	The model demonstrated an accuracy of 95%.
6	Pradhan et al. [17]	2020	Artificial neural network	With 70% training data and 30% testing data, the model achieved an accuracy of 85.09%
7	Kannadasan et al. [18]	2019	Deep Neural Network (DNN)	The results demonstrated that the classifier achieved an accuracy of 86.26%.
8	Maniruzzaman et al. [22]	2017	Linear Discriminant Analysis (LDA), Quadratic Discriminant Analysis (QDA), and Naive Bayes (NB)	The results demonstrated that the model achieved an accuracy of 81.97%.
9	Azad et al. [19]	2021	Synthetic Minority Over-sampling Technique (SMOTE), Genetic Algorithm (GA), and Decision Tree (DT)	The model was tested on the Pima Indians Diabetes (PID) dataset and achieved an accuracy of 82.1256%.
10	Kumari et al. [20]	2021	k-Nearest Neighbours (kNN)	The model achieved an accuracy of 92.28% on the diabetes dataset
11	Abokhzam et al. [21]	2021	Machine Learning grid-based Random Forest	The model achieved an accuracy of 95.7%.

### 2.2. Fuzzy Logic for Diabetes Detection

Table 2 summarizes all of the FL diabetes detection techniques discussed in this section. Siva et al. [23] proposed a model for diabetes prediction that incorporated fuzzy rules and the grey wolf optimization (GWO) algorithm. The Pima Indians Diabetes (PID) dataset was used by the researchers in this study. Firstly, 17 fuzzy rules were generated from the selected dataset. Afterward, fuzzy rules were optimized using the grey wolf optimization (GWO) algorithm. The classification was carried out by using these optimal rules. The proposed model obtained an accuracy of 81%. Using fuzzy logic, Cheruku et al. [24] suggested a system called RST-BatMiner for diabetes prediction. Rough set theory (RST) and the bat optimization algorithm (BA) were used to generate comprehensible fuzzy rules. The proposed system consisted of two stages. In the first stage, it incorporated the RST for optimal feature selection. In the second stage, it integrated BA and boosting algorithms to generate accurate fuzzy rules. The model was evaluated on the diabetes dataset, and it gave 85.33% accuracy. The proposed model exhibited great accuracy in early diabetes detection compared to previous algorithms.

Singh et al. [25] proposed a novel fuzzy rule miner (ANT FDCSM). The rule miner used an ant colony meta-heuristic for the prediction of diabetes. To compute the heuristic knowledge, a hybrid node split measure (SW FDCSM) was employed. A diabetes dataset was used to evaluate the performance of ANT FDCSM using 10-fold cross-validation. The results demonstrated 87.7% accuracy, 92.2% sensitivity, and 80.3% specificity. Lukmanto et al. [26] proposed a model for the early detection of diabetes. The feature selection technique was used to get the optimal features from the dataset. Afterward, a support vector machine (SVM) was used to generate optimal fuzzy rules. PID dataset was chosen to evaluate the performance of the entire system. The results demonstrated that the system achieved an accuracy of 89.02% in predicting diabetes.

Sharma et al. [27] proposed a novel technique for the prediction of diabetes. Features were extracted from the PID dataset and then used as input variables. Afterward, the mediative fuzzy logic (MFL)-based inference method was applied to diagnose diabetes. Furthermore, an algorithm was proposed that was based on MFL. In this study, approximately 150 rules were generated using the proposed algorithm, but only 28 rules were chosen because they showed drastic changes in the results. Then, these rules were used to diagnose diabetes. Thungrut et al. [28] proposed a method based on the fuzzy genetic algorithm for the classification of diabetes. To improve classification accuracy, two algorithms that were made up of fuzzy algorithms and genetic algorithms were employed. Furthermore, the synthetic minority over-sampling technique (SMOTE) was employed to tackle the ambiguity in the dataset. The experiments demonstrated that five-fold cross-validation was a suitable technique to measure the performance of the proposed research. The system showed 87.40% accuracy, 86.82% sensitivity, and 88% specificity. Zhang et al. [29] proposed a parallel ensemble fuzzy classifier FP-TSK-FW for diabetes detection. Parallel-based fuzzy partition, fuzzy weighted ensemble, and Takagi-Sugeno-Kang were used to construct the classifiers. Firstly, the fuzzy clustering algorithm FCM was used to partition the training dataset. Afterward, several TSK-fuzzy sub-classifiers were constructed using training data. All these sub-classifiers were generated in parallel and with varied structures. Lastly, the final prediction of the proposed system is carried out using the fuzzy weight of each classifier. The proposed ensemble classifier experimented on the PID dataset. The finding demonstrated that the FP-TSK-FW is effective in the classification of diabetes.

Mujawar et al. [30] proposed a fuzzy rule-based expert system (WebFESDD) that incorporated a web facility to diagnose diabetes. The PID dataset was used to evaluate the performance of the proposed expert system. The results demonstrated 84% prediction accuracy. The limitation of the proposed expert system was it focused on a specific age group. The system could have been further improved by including different age groups. Chen et al. [31] proposed a method based on the Takagi-Sugeno-Kang (TSK) fuzzy rule for the diagnosis of diabetes. The proposed method began with the creation of a crisp rule base using a decision tree, a mechanism capable of learning fundamental rules that represent the relationships between domain input and output attributes with low overhead. Afterward, the crisp rule base was converted to the fuzzy rule base using Gaussian membership functions (MF). Then the fuzzy rule base was inputted to the neuro-fuzzy framework that enhanced the rules. The proposed method was implemented and tested using the PID dataset and gave 75.67% accuracy.

Mansourypoor et al. [10] proposed a novel fuzzy rule-based system (FRBS) based on reinforcement learning (RL), called reinforcement learning-based evolutionary fuzzy rule-based system (RLEFRBS), for the diagnosis of diabetes. Initially, a rule base (RB was generated from numerical data and then optimized. After that, unnecessary rules were discarded using confidence measures. Furthermore, redundant conditions in the antecedent parts were cut down. Lastly, a final RB was constructed using the genetic algorithm (GA), and it consisted of a subset of rules initially developed using numerical data. Afterward, membership functions were tuned, and the weights were adjusted using reinforcement learning (RL) to increase RLEFRBS performance. In addition, RLEFRBS used an efficient rule stretching mechanism to cope with uncovered instances. Two datasets were used to test RLEFRBS performance: the PID dataset and Biosat diabetes dataset, and these datasets gave 82.5% and 96.5% accuracy, respectively. Vaishali et al. [32] combined the genetic algorithm and multiple objective evolutionary fuzzy classifier for the prediction of diabetes. These two techniques were combined to achieve better prediction accuracy. At first, the PID dataset was selected. Next, Goldberg’s genetic algorithm was applied to reduce the features of the dataset. This algorithm minimizes the features and maximizes the classification rate. Lastly, the performance of the multi-objective evolutionary (MOE) fuzzy classifier was checked using both original and feature-reduced datasets. With 70% training and 30% testing data, the classifier achieved an accuracy of 83.0435%. Geman et al. [33] developed a hybrid adaptive neuro-fuzzy inference system (ANFIS). The researchers used the PID dataset. Afterward, ANFIS was integrated with the diabetes pedigree function. A genetic relationship was used to define the fuzzy rule base with multiple premises. The proposed method was implemented using ANFIS fuzzy logic toolbox and MATLAB. The proposed system demonstrated an accuracy of 85.35% for training data and 84.27% for testing data.

Bhuvaneswari et al. [34] proposed a novel system for the prediction of diabetes. The proposed system combined temporal feature selection and temporal fuzzy ant miner tree (TFAMT) classifier for effective diabetes prediction. A novel temporal weighted genetic algorithm was used that preprocessed the imagery and textual data. Furthermore, the intelligent fuzzy rules were generated from the weighted temporal capability of TFAMT. Afterward, the fuzzy rules were optimized. The proposed system was tested using the UCI diabetes and the retinopathy image datasets and gave 83.7% accuracy. Deshmukh et al. [35] developed a hybrid fuzzy deep learning approach for the detection of diabetes. Firstly, the data was fuzzified. After that, a 5 × 5 fuzzy matrix was constructed where columns represented features of the dataset, while rows represented the fuzzy value of the features. Lastly, the fuzzy matrix was fed into the convolution neural network (CNN). In this research, the three experiments were carried out on the diabetes dataset. Two experiments were conducted on neural network (NN) and one on CNN. The results demonstrated that the fuzzified CNN approach outperformed the traditional NN approach and achieved an accuracy of 95%.

### 2.3. Fuzzy Logic and Machine Learning Techniques for Other Diseases

Orujov et al. [36] created a blood vessel identification method using contour detection and Mamdani fuzzy rules. The algorithm was tested on three different datasets and achieved accuracies of 0.865, 0.939, and 0.950, respectively. The suggested method employed linguistic threshold criteria, which made it superior to existing strategies. Fatema et al. [37] developed a distributed type 2 FL (DT2FL) method and employed ML-based mobile agents to implement it. The paper concentrated on the DT2FL application for analyzing MRI data. The flexibility of DT2FL with ML models makes it appropriate for the healthcare industry.

Reddy et al. [38] developed a model based on adaptive GA and FL (AGAFL) for the early identification of heart problems. The model was made up of two modules: RS-based feature selection and FRB-based classification. The AGA was used to determine the rules produced by fuzzy classifiers. The UCI heart disease datasets were used to evaluate the performance of the proposed model. Experimental research demonstrated that the proposed technique outperformed currently available approaches. Singla et al. [39] proposed an FES for kidney diseases detection. To evaluate the performance of the proposed system, 80 tests were performed on the FES. The outputs of 80 tests were compared with the predicted output. This system succeeded in 93.75% of the tests. The FES was developed using MATLAB.

Khalil et al. [40] developed a fuzzy soft expert system for the detection of lung cancer. A fuzzy membership function and some algorithms were used to build the system. The system was comprised of different steps. At first, input was converted to fuzzy numbers. In the next step, a fuzzy set was developed using the fuzzy numbers. Afterward, the fuzzy set was reduced using the reduction method. Lastly, the proposed algorithm was used to generate the output. The system was tested on 45 patients and achieved 100% accuracy in predicting lung cancer. Luo et al. [41] developed a self-supervised model that employed fuzzy clustering. Three modules were developed: feature learning, reconstruction, and fuzzy self-supervision. These modules were used to generate training guidance for the whole network. To assess the efficiency of the proposed model, three retinal datasets were used, and the results showed that the proposed model attained the highest accuracy of 82.8%.

## 3. Materials and Methods

Fuzzy rule-based systems are systems in which crisp data is transformed into fuzzy sets. This process is called fuzzification. Afterward, fuzzy inference techniques (Mamdani and Sugeno) are applied to construct fuzzy rules. Based on the fuzzy rules, the output is derived.

### 3.1. Dataset

The dataset (https://www.kaggle.com/uciml/pima-indians-diabetes-database (accessed on 27 November 2021)) that has been used for this study is taken from Kaggle, which is an online dataset database. This dataset is included several attributes through which we have predicted whether the patient can get diabetes or not. All the instances of the dataset are women and at least 21 years old. The dataset is comprised of 768 patients, out of which 268 samples are identified as diabetic while 500 samples are identified as non-diabetic. The dataset is included nine attributes that are as follows: the number of pregnancies, plasma glucose concentration, diastolic blood pressure, serum insulin, body mass index (BMI), triceps, skinfold thickness, diabetes pedigree function, age, and a class variable. The other eight attributes, on the other hand, are features variables and are independent variables. There are just two values in the class variable: Yes and No, with ‘Yes’ indicating diabetic and ‘No’ indicating non-diabetic. Table 3 presents more detailed information about each parameter of the dataset.

### 3.2. Data Pre-Processing

**Figure 1:**
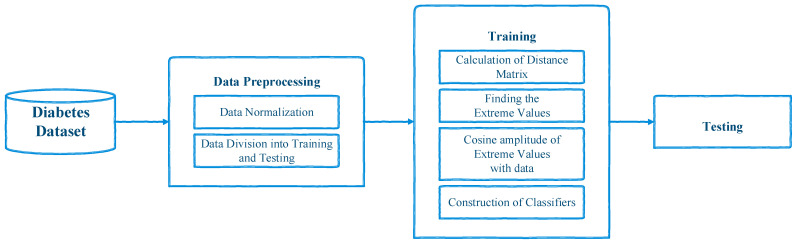
The framework of the proposed system.

This section describes how the data is pre-processed in our proposed method. Data pre-processing helps in generating a reliable classification model that provides high accuracy. Therefore, the data has been normalized and it ranges from 0 to 1. At first, data is categorized into two parts: class 0 and class 1. Healthy people are represented by class 0, while sick people are represented by class 1. Afterward, two matrices have been created. Let A_0_ and A_1_ be the matrices containing data of class 0 and class 1, respectively. Where A_0_ ∈ $R^{n\times k}$ and A_1_ ∈ $R^{m\times k}$, k is the size of each sample, and there are n and m number of samples for class 0 and class 1, respectively. In data normalization, all feature variables or independent variables of the dataset are rescaled from 0 to 1. As a result, the attribute’s maximum value is 1, and its smallest value is 0. The normalized $\underline{\hat{x}}$ of $\;\underline{x}\;$ is given below:

(1) $$\underline{\hat{x}}=\frac{\;\underline{x}\;}{\max\left(\underline{x}\right)}$$

where $\underline{x}$ is a vector that contains an instance of a dataset. This normalizing process is used to transform the data into a fuzzy set. Figure 1 illustrates the framework of the proposed system.

### 3.3. Classification

After performing normalization on the attributes of the dataset, training, and testing are performed. The dataset is sliced into training and testing parts using linear sampling. Table 4 presents more information about the dataset division. 52% of data is used for training, while 48% of data is used for testing. In this work, two fuzzy classifiers are constructed to predict diabetes.

The major benefit of the proposed work is it finds the degree of belongingness for each instance of the dataset. Afterward, based on the degree of belongingness, a person is classified as diabetic or non-diabetic. To perform classification, firstly, the distance matrix has been determined. The training data has been used to find the distance matrix D using Euclidean distance. The equation for the distance matrix is as follows:

(2) $$D=\left[\begin{array}{ccc} d_{11} & \cdots & d_{1m}\\ \vdots & \ddots & \vdots \\ d_{n1} & \cdots & d_{\mathrm{nm}} \end{array}\right]$$

where d_ij_ is the instance of the D matrix. The equation for d_ij_ is given below:

(3) $$d_{\mathrm{ij}}={\left||{\underline{x}}_{i}\;-\;{\underline{y}}_{j}|\right|}_{2}$$

where x_i_ is the ith row of A_0_ and y_j_ is the jth row of A_1_, i ∈ [1, n] and j ∈ [1, m]. Using the distance matrix, extreme examples are found. Such as d = max d_ij_. Let d is the pth row and qth column of D. Therefore, the extreme examples are ${\underline{x}}_{p}$ and ${\underline{y}}_{q}$. Afterward, the cosine amplitude method has been used to find similarities of the entire data with extreme examples. Then, based on each extreme example, two classifiers have been constructed.

#### 3.3.1. Classifier 1

In classifier 1, the cosine amplitude of the data is calculated with ${\underline{x}}_{p}$. Therefore, the dot product of matrix A_0_ is taken with vector ${\underline{x}}_{p}^{T}$. Similarly, the dot product of matrix A_1_ is taken with vector ${\underline{x}}_{p}^{T}$. As a result, two vectors ${\underline{U}}_{0}$ and ${\underline{U}}_{1}$ have been created. The equations are given below for class 0 and class 1, respectively:

(4) $${\underline{U}}_{0}=A_{0}\;\cdot \;{\underline{x}}_{p}^{T}$$

(5) $${\underline{U}}_{1}=A_{1}\;\cdot \;{\underline{x}}_{p}^{T}$$

It is observed that ${\underline{U}}_{0}$ and ${\underline{U}}_{1}$ are not fuzzy sets. Therefore, ${\underline{U}}_{0}$ and ${\underline{U}}_{1}$ are normalized. Equations (6) and (7) demonstrate the normalization of ${\underline{U}}_{0}$ and ${\underline{U}}_{1}$. While $\sum {\underline{U}}_{0}\;$ and $\sum {\underline{U}}_{1}$ are the summation of all values in the vector ${\underline{U}}_{0}$ and ${\underline{U}}_{1}$, respectively. Where $\sum {\underline{U}}_{0}\;$ and $\sum {\underline{U}}_{1}$ are defined in Equations (8) and (9). In these equations, 1 is a vector with all values as one.

(6) U_^0=αU_0∑U_0

(7) U_^1=αU_1∑U_1

(8) $$\sum {\underline{U}}_{0}={\underline{U}}_{0}^{T}\;\times \;1\;\;\mathrm{where}\;1\;\in \;R^{n}$$

(9) $$\sum {\underline{U}}_{1}={\underline{U}}_{1}^{T}\;\times \;1\;\;\mathrm{where}\;1\;\in \;R^{m}$$

Further, a scalar α has been multiplied to normalize the values on a large scale. However, α does not affect classification. Now, elements appearing in U_^0 and U_^1 are considered as unordered series, and histograms h_0_(z) and h_1_(z) are computed where z ∈ R. Note that h_0_(z) and h_1_(z) are continuous fuzzy sets. λ-cut has been applied on h_1_(z), say, h_1λ_(z). The set h_1λ_(z) defines regions for class 1.

#### 3.3.2. Classifier 2

In classifier 2, the cosine amplitude of the data is calculated with ${\underline{y}}_{q}$. The equations are given below for class 0 and class 1, respectively:

(10) $${\underset{\_}{V}}_{0}=A_{0}\;\cdot \;{\underline{y}}_{p}^{T}$$

(11) $${\underline{V}}_{1}=A_{1}\;\cdot \;{\underline{y}}_{p}^{T}$$

It is observed that ${\underline{V}}_{0}$ and ${\underline{V}}_{1}$ are not fuzzy sets. Therefore, ${\underline{V}}_{0}$ and ${\underline{V}}_{1}$ are normalized in Equations (12) and (13):

(12) V_^0=βV_0∑V_0

(13) V_^1=βV_1∑V_1

where $\sum {\underline{V}}_{0}$ and $\sum {\underline{V}}_{1}$ is the summation of all values in the vector ${\underline{V}}_{0}$ and ${\underline{V}}_{1}$. Equations (14) and (15) define $\sum {\underline{V}}_{0}$ and $\sum {\underline{V}}_{1}$, while 1 is a vector with all values set as one.

(14) $$\sum {\underline{V}}_{0}={\underline{V}}_{0}^{T}\;\times \;1\;\;\mathrm{where}\;1\;\in \;R^{n}$$

(15) $$\sum {\underline{V}}_{1}={\underline{V}}_{1}^{T}\;\times \;1\;\;\mathrm{where}\;1\;\in \;R^{m}$$

A scalar β has been multiplied to normalize the values on a large scale, and it does not affect the classification. Now, elements appearing in V_^0 and V_^1 are considered as unordered series, and histograms l_0_(z) and l_1_(z) are computed where z ∈ R. Note that l_0_(z) and l_1_(z) are continuous fuzzy sets. λ-cut has been applied on l_0_(z), say, l_0λ_(z). The set l_0λ_(z) defines regions for class 0.

Now classification is performed: Let $\underline{x}$ be an input vector and is classified whether healthy or sick (class 0 and class 1, respectively). As x_i_ and y_j_ are computed in Equation (3):

If ${\underline{x}}^{T}$${\underline{x}}_{p}$ ∈ h_1λ_(z), then $\underline{x}$ belongs to class 1.

If ${\underline{x}}^{T}$${\underline{y}}_{q}$ ∈ l_0λ_(z), then $\underline{x}$ belongs to class 0.

## 4. Results and Discussion

**Figure 2:**
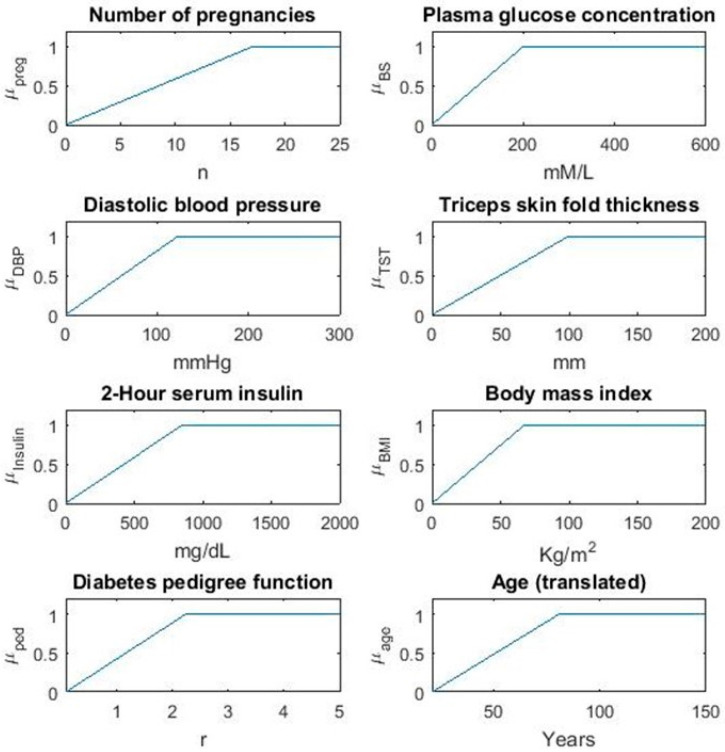
Fuzzy membership values for variables.

This section presents a detailed analysis of the proposed fuzzy model for diabetes prediction. The proposed model has been evaluated using a diabetes dataset taken from an online database, Kaggle, and implemented on MATLAB R2021a (version 10.0). 52% of data has been used for training and 48% has been of data is used for testing. At first, the dataset has been normalized, which means that each numerical value in the dataset is between 0 and 1. The equation for normalization is given in Equation (1). Fuzzy membership values for the variables considered in this study are shown in Figure 2, versus the respective universes of discourse.

**Figure 3:**
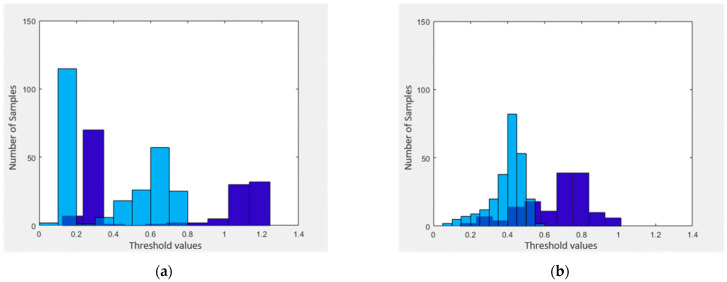
Demonstrates the threshold values of both classifiers for the training phase. (**a**) shows the threshold values for classifier 1 and (**b**) shows the threshold values for classifier 2.

Moreover, the cosine amplitude method has been used to find the thresholds for classifier 1 and classifier 2. Graphical representation of thresholds for the training phase is shown in Figure 3.

**Figure 4:**
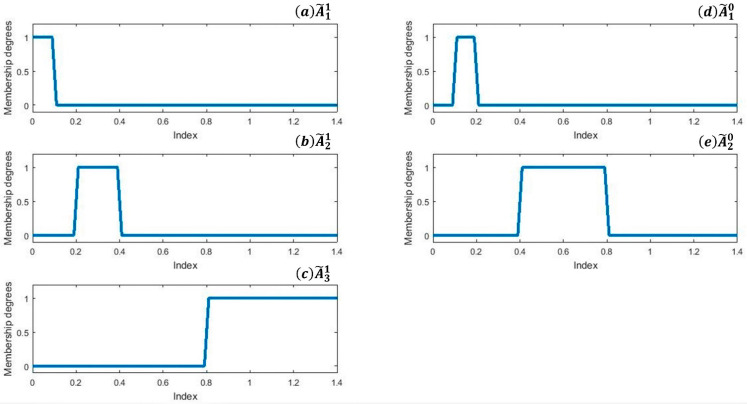
MFs. (**a**–**c**) shows MFs for class 1 while (**d**,**e**) shows MFs for class 2.

With the information perceived from Figure 3a, we find corresponding threshold values in the interval [0, 1.4] listed as [0.2, 0.4, 0.8]. Based on these thresholds, we set up three fuzzy MFs namely ${\tilde{A}}_{1}^{},\;{\tilde{A}}_{2}^{},\;\mathrm{and}\;{\tilde{A}}_{3}^{}$ for sickness and two membership functions ${\tilde{A}}_{1}^{0}\;\mathrm{and}\;{\tilde{A}}_{2}^{0}$ for health shown in Figure 4.

As in Equation (16) ${\tilde{A}}_{1}^{},\;{\tilde{A}}_{2}^{},\;\mathrm{and}\;{\tilde{A}}_{3}^{}\;$ are representing MFs for sickness, we established a single MF by aggregation through their unions:

(16) $${\tilde{A}}_{}^{1}={\tilde{A}}_{1}^{1}\cup {\tilde{A}}_{2}^{1}\cup {\tilde{A}}_{3}^{1}$$

Similarly for healthy status aggregate is calculated by using Equation (17):

(17) $${\tilde{A}}_{}^{0}={\tilde{A}}_{1}^{0}\cup {\tilde{A}}_{2}^{0}$$

For a given instance *x*, ($\mu_{{\tilde{A}}^{0}}\left(x\right),\mu_{{\tilde{A}}^{1}}\left(x\right))$ defines fuzzy grades of the health status of *x*, whereas the $\mu_{{\tilde{A}}^{0}}\left(x\right)\;\mathrm{and}\;\mu_{{\tilde{A}}^{1}}\left(x\right)$ represent the status of healthiness and sickness level respectively. Rules for classifier 1 are mentioned below.

If $\mu_{{\tilde{A}}^{0}}\left(x\right)\geq \;\mu_{{\tilde{A}}^{1}}\left(x\right)$ then “x is healthy”

If $\mu_{{\tilde{A}}^{0}}\left(x\right)<\mu_{{\tilde{A}}^{1}}\left(x\right)$ then “x is sick“

**Figure 5:**
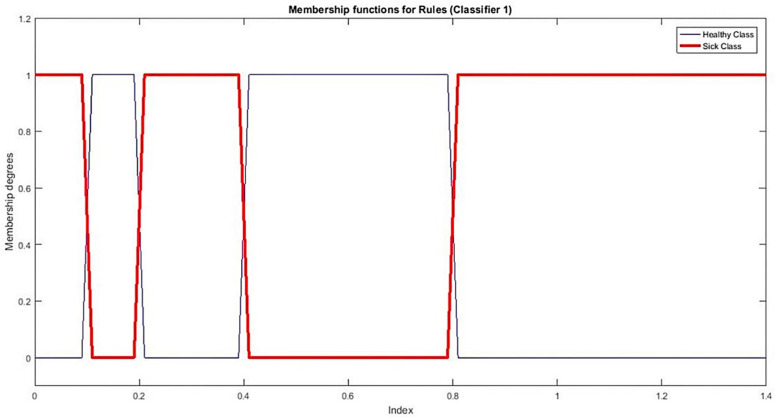
MFs for classifier 1.

With the information perceived from Figure 3b, we find a threshold 0.5. Based on this threshold value, we setup MFs namely ${\tilde{B}}_{}^{0}\;\mathrm{and}\;{\tilde{B}}_{}^{1}$ for sickness status respectively. MFs for classifier 1 are shown in Figure 5.

**Figure 6:**
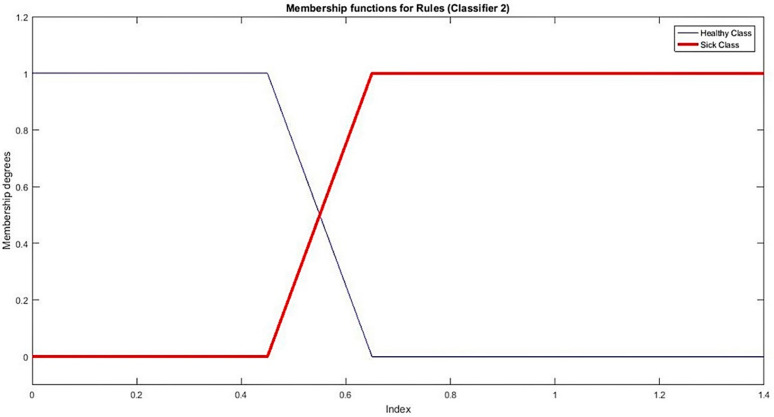
MFs for classifier 2.

Rules for classifier 2 are as under and MFs for classifier 2 are shown in Figure 6.

If $\mu_{{\tilde{B}}^{0}}\left(x\right)\geq \;\mu_{{\tilde{B}}^{1}}\left(x\right)$ then “x is healthy”

If $\mu_{{\tilde{B}}^{0}}\left(x\right)<\mu_{{\tilde{B}}^{1}}\left(x\right)$ then “x is sickness”

Afterward, these rules have been employed to classify the dataset. A confusion matrix has been constructed to see the results of our classifiers. The confusion matrix is given in Table 5. TN which stands for true negatives is a measure of the number of instances that are non-diabetic and classified as non-diabetic, while, FP, false positive is a measure of the number of instances where a patient is non-diabetic and classified as diabetic. FN which stands for false negatives is a measure of the number of instances that are diabetic and classified as non-diabetic. TP, true positive is a measure of the number of instances that are diabetic and classified as diabetic. The testing results of both classifiers are demonstrated using a confusion matrix in Table 6. Classifiers 1 and 2 is made a total of 368 predictions. Out of these 368 predictions, classifier 1 predicted “yes” 121 times and “no” 247 times. The predictions of classifier 1 include 113 TP, 5 FN, 8 FP, and 242 TN, while classifier 2 predicted “yes” 136 times and “no” 232 times. The predictions of classifier 2 include 106 TP, 12 FN, 5 FP, and 245 TN. While in reality, 118 samples are diabetic, and 250 samples are non-diabetic.

The two classifiers have been evaluated using four key criteria: classification accuracy or classification rate, precision, recall, and F-measure. The classification accuracy is obtained using the following formula:

(18) $$\mathrm{Classification}\;\mathrm{accuracy}=\frac{\mathrm{TP}+\mathrm{TN}}{\mathrm{TP}+\mathrm{TN}+\mathrm{FN}+\mathrm{FP}}\;$$

Our proposed classifiers’ accuracies have been compared to that of other well-known fuzzy classification methods in Table 7. The comparison is demonstrated that the proposed classifiers have outperformed the state-of-the-art fuzzy classification techniques in classification accuracy. Table 7 compares our results with existing fuzzy rule-based systems, fuzzy genetic algorithms, fuzzy CNN, and other fuzzy techniques. Our proposed classifiers demonstrate the classification accuracy of 96.47% and 95.38%, respectively. This means that the proposed fuzzy classifiers would be extremely successful in detecting diabetes.

After calculating classification accuracy, the recall, precision, and f-measure of the classifiers have been calculated. Table 8 shows the values of the accuracy, precision, recall, and F-measure for testing. The formulas for recall, precision, and f-measure are given below:

(19) $$\mathrm{Recall}=\frac{\mathrm{TP}}{\mathrm{TP}+\mathrm{FN}}$$

(20) $$\mathrm{Precision}=\frac{\mathrm{TP}}{\mathrm{TP}+\mathrm{FP}}$$

(21) $$F-\mathrm{measure}=\frac{2\;\times \;(\mathrm{Recall}\;\times \;\mathrm{Precision})}{\mathrm{Recall}+\mathrm{Precision}}$$

Both classifiers have shown good results by using parameters like accuracy, precision, recall, and F-measure. Classifier 1 is achieved 96.47%, 95.76%, 93.39%, and 94.56% scores for accuracy, recall, precision, and F-measure, respectively, while classifier 2 achieved 95.38%, 89.83%, 95.50%, and 92.58% scores for accuracy, recall, precision, and F-measure, respectively.

## 5. Conclusions

Diabetes has recently emerged as a major public health problem. Diabetes is a currently incurable disease that can lead to a variety of serious complications that endanger the health of diabetic patients. Therefore, early diagnosis of diabetes is very crucial to control and prevent its impact on health. For the early detection of diabetes, a variety of approaches have been proposed by researchers. In this paper, a fuzzy rule-based system for the early prediction of diabetes has been proposed and implemented. Two fuzzy classifiers have been constructed which classify either a person diabetic or non-diabetic. First, a distance matrix was constructed using Euclidean distance, and the maximum value of the matrix was determined. Second, cosine amplitude was used to determine belongingness. This degree of belongingness helps in the classification of diabetes. Afterward, fuzzy rules based on the two classifiers have been developed. Lastly, classification accuracy, precision, recall, and f-measure are used as performance parameters. To evaluate the performance of classifiers, a diabetes dataset has been used. Classifiers 1 and 2 have demonstrated 96.47% and 95.38% accuracy, respectively. The findings indicate that the proposed model can accurately predict diabetes at an early stage. In the future, the proposed model will be used to diagnose other diseases.

## Figures and Tables

**Table 2:** Summary of fuzzy logic techniques for diabetes detection.

Sr. No.	Reference	Year	Methodology	Finding and Results
1	Siva et al. [23]	2019	Fuzzy rules and grey wolf optimization (GWO) algorithm	The classification was carried out by using optimal rules. The proposed model obtained an accuracy of 81%.
2	Cheruku et al. [24]	2018	Rough Set Theory (RST) and the Bat Optimization Algorithm	The system gave an accuracy of 85.33%.
3	Singh et al. [25]	2019	Fuzzy rule miner (ANT FDCSM).	The results demonstrated 87.7% accuracy, 92.2% sensitivity, and 80.3% specificity.
4	Lukmanto et al. [26]	2019	Fuzzy support vector machine	The results demonstrated that the system achieved an accuracy of 89.02% in predicting diabetes.
5	Sharma et al. [27]	2021	Mediative Fuzzy Logic	The researchers generated optimal fuzzy rules for diabetes prediction.
6	Thungrut et al. [28]	2019	Fuzzy genetic algorithm	The system showed 87.40% accuracy, 86.82% sensitivity, and 88% specificity.
7	Zhang et al. [29]	2019	Parallel ensemble fuzzy classifier	The finding demonstrated that the FP-TSK-FW is effective in the classification of diabetes.
8	Mujawar et al. [30]	2019	Fuzzy expert system	The results demonstrated 84% prediction accuracy.
9	Chen et al. [31]	2019	Neuro-fuzzy	The proposed model gave 75.67% accuracy on the selected dataset.
10	Mansourypoor et al. [10]	2017	Fuzzy rule-based system	The researchers used two datasets to test RLEFRBS performance: the Pima Indian Diabetes dataset and the Biosat Diabetes dataset, and these datasets gave 82.5% and 96.5% accuracy, respectively.
11	Vaishali et al. [32]	2017	Multiple objective evolutionary fuzzy classifier	With 70% training and 30% testing data, the classifier achieved an accuracy of 83.0435%.
12	Geman et al. [33]	2017	Adaptive Neuro-Fuzzy Inference System	The proposed system demonstrated accuracy for training data is 85.35%, and testing data is 84.27%.
13	Bhuvaneswari et al. [34]	2018	Temporal fuzzy ant miner tree	The proposed system achieved an 83.7% accuracy.
14	Deshmukh et al. [35]	2018	Fuzzy CNN	The results demonstrated that the fuzzified CNN approach outperformed the traditional NN approach and achieved an accuracy of 95%.

**Table 3:** Description of the dataset attributes.

Sr. No.	Parameters of Dataset	Description of Parameters	Normal Range
1	Number of pregnancies	Number of times the person gets pregnant	0–17
2	Plasma glucose concentration	Represents the concentration of glucose in a person’s body.	0–199
3	Diastolic blood pressure	Represents the diastolic blood pressure in (mm Hg)	0–122
4	Triceps skinfold thickness	Represents triceps skinfold thickness in (mm)	0–99
5	Serum insulin	Represent 2 h serum insulin in (µU/mL)	0–846
6	Body mass index	It is a value derived from the weight and height of a person (weight in kg/(height in m)^2^).	0–67.1
7	Diabetes pedigree function	It represents the history of diabetes associated with a particular person.	0.0078–2.42
8	Age	Age of the person in years	21–81
9	Class variable	It represented two classes: diabetic and non-diabetic	Yes/No

**Table 4:** Dataset division into training and testing.

	Class 0	Class 1
**Total**	500	268
Training	250	150
Testing	250	118

**Table 5:** Description of the confusion matrix.

	Predicted Yes	Predicted No
**Actual Yes**	True Positive (TP)	False Negative (FN)
**Actual No**	False Positive (FP)	True Negative (TN)

**Table 6:** Confusion matrix for both fuzzy classifiers.

		Predicted Yes	Predicted No
Classifier 1	**Actual Yes**	113	5
	**Actual No**	8	242
Classifier 2	**Actual Yes**	106	12
	**Actual No**	5	245

**Table 7:** Comparison of our fuzzy classifiers with other fuzzy techniques.

Sr. No.	Reference	Methods	Classification Accuracy
1	Siva et al. [23]	Fuzzy rules and grey wolf optimization (GWO) algorithm	81%
2	Cheruku et al. [24]	Rough Set Theory (RST) and the Bat Optimization Algorithm	85.33%
3	Singh et al. [25]	Fuzzy rule miner (ANT FDCSM).	87.7%
4	Lukmanto et al. [26]	Fuzzy support vector machine	89.02%
5	Thungrut et al. [28]	Fuzzy genetic algorithm	87.40%
6	Mujawar et al. [30]	Fuzzy expert system	84%
7	Chen et al. [31]	Neuro-fuzzy	75.67%
8	Mansourypoor et al. [10]	Fuzzy rule-based system	82.5% and 96.5%
9	Vaishali et al. [32]	Multiple objective evolutionary fuzzy classifier	83.0435%
10	Geman et al. [33]	Adaptive Neuro-Fuzzy Inference System	For training data, 85.35% and testing data, is 84.27%
11	Bhuvaneswari et al. [34]	Temporal fuzzy ant miner tree	83.7%
12	Deshmukh et al. [35]	Fuzzy CNN	95%
13	Fuzzy classifier 1	Fuzzy	96.47%
14	Fuzzy classifier 2	Fuzzy	95.38%

**Table 8:** The performance measures for both classifiers.

	Performance Measure	Percentage
Classifier 1	Accuracy	96.47%
	Recall	95.76%
	Precision	93.39%
	F-measure	94.56%
Classifier 2	Accuracy	95.38%
	Recall	89.83%
	Precision	95.50%
	F-measure	92.58%

## Data Availability

We have used only publicly available dataset for experimentation. The dataset is available at: https://www.kaggle.com/uciml/pima-indians-diabetes-database (accessed on 27 November 2021).
